# Post typhoid fever neuroretinitis with serous retinal detachment and choroidal involvement-A case report

**DOI:** 10.1016/j.ajoc.2021.101025

**Published:** 2021-01-30

**Authors:** Anadi Khatri, Bivek Wagle, K.C. Hony, Babu Dhanendra Chaurasiya, Satish Timalsena, Kinsuk Singh, Rupesh Agrawal

**Affiliations:** a Department of Vitreoretinal Services, Birat Eye Hospital, Biratnagar, Nepal; b Department of Ophthalmology, Birat Medical College and Teaching Hospital, Biratnagar, Nepal; c Morehouse School of Medicine, Atlanta, GA, USA; d Department of Ophthalmology,Birat Eye Hospital, Biratnagar, Nepal; e Department of Ophthalmology, Narayani Hospital, Narayani, Nepal; f Vasan Eye Care, Mysuru, Karnataka, India; g National Healthcare Group Eye Institute, Tan Tock Seng Hospital, Singapore; h Singapore Eye Research Institute, Singapore; i Moorfields Eye Hospital, NHS Foundation Trust, London, UK

**Keywords:** Salmonella typhi, Typhoid fever, Vasculitis, OCT, OCTA, Choroid, Neuroretinitis,Serous retinal detachment

## Abstract

**Purpose:**

To report post typhoid fever neuroretinitis with Serous Retinal Detachment and choroidal involvement.

**Observation:**

Patients with diminished vision post typhoid fever can present with neuroretinitis with serous retinal detachment.

**Conclusion and importance:**

With help from noninvasive imaging such as optical coherence tomography angiography(OCTA) and Deep Range Imaging(DRI), we were able to conclude choroidal involvement – which has not been discussed in literatures yet.OCTA and choroidal thicknessboth served as agood indicators for monitoring the response of treatment in this case.

## 1 Introduction

*Salmonella typhi* is a common gastrointestinal pathogen. It causes typhoid—a waterborne infectious disease.^1^ However,on rare instances,the organism has been reported to cause ocular pathologies, ranging fromuveal complications such as iritis, retinal hemorrhage, choroiditis, endophthalmitis and panophthalmitis to retinal complications such as retinitis and vasculitis.^2^ The hypothesized mechanism is either via a direct invasion by the organism or via an immune-complex mediated hypersensitivity reaction.^3^

Here, we report a patient who presented with neuroretinitiswithserous retinal detachment following a typhoid feverfour weeks prior to presentation.

## 2 Case report

A 21-year-old femalepresented at our retina clinic, complaining of sudden onset painless diminution of vision in her left eye since 27 days. After seven days of the onset, she started experiencing the same symptoms on her right eye.She initially visited a local eye clinic, from where she was referred to our comprehensive care center.On questioning, she has a recent history of typhoid fever, onset approximately 45 days ago and resolved after taking oral azithromycin 500mg and cefixime 400mg BID for 10 days. Diagnosis was established by both blood culture a positive Widal test showing *S. typhi* O antigen titer at 1:160 and H antigen at 1:80. Her AH and BH antigens were negative.

**Fig. 1:**
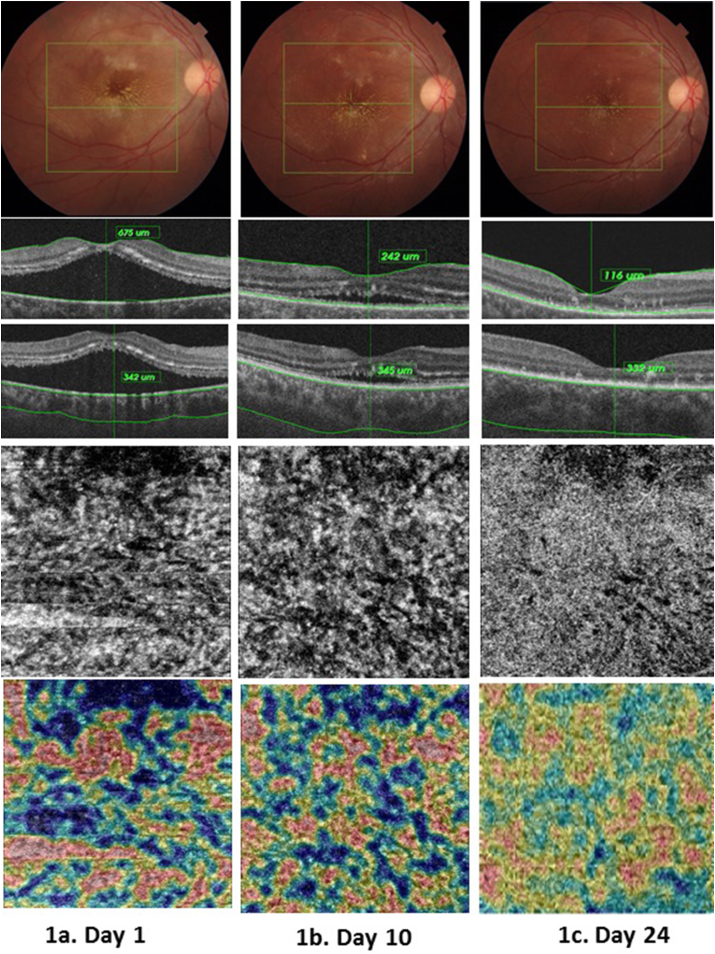
Response of right eye to treatment. 1a. Day 1 (Top)Fundus photo showing macular star with cotton wool spots and hard exudates in the posterior pole. (Middle) Central serous retinal detachment with increased choroidal thickness. (Lower) OCTA of choriocapillaris showing flow voids and coarse pattern. (Bottom) Vascular density mapping of the choroidal vascular pattern showing the areas of flow and voids as in a heat map. 1b. Day 10 (Top) Fundus photos showing decreased areas of cotton wool spots.(Middle) Decreased sub retinal fluid level but choroidal thickness remained unchanged. (Lower) Choriocapillaris signals returning to normal – a more granular pattern.(Bottom)Vascular density mapping of the choroid showing returning of the vascular pattern 1c. Day 24(Top) Resolving macular star.(Middle) Complete resolution of subretinal fluid with decreased macular thickness with normalization of choroidal thickness. (Lower and Bottom) OCTA and vascular density map of choroid showing near normal choriocapillaris.

**Fig. 2:**
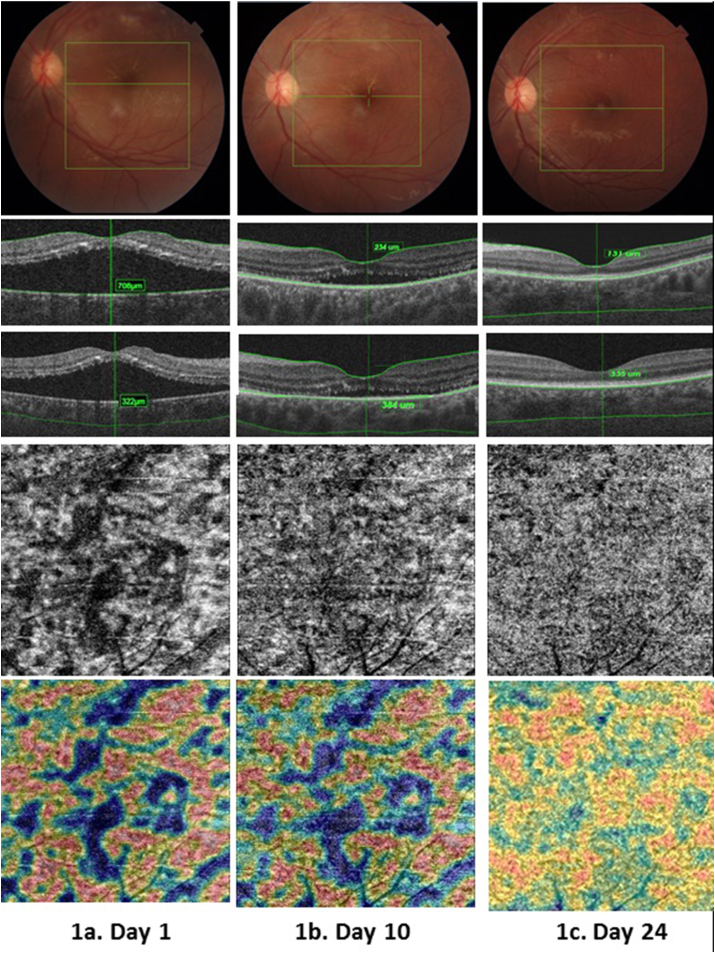
Response of left eye to treatment.2a. Day 1 (Top)Left eye had less numbers of cotton wool spot and an incomplete macular star. (Middle) Central serous retinal detachment was with increased choroidal thickness. (Lower and bottom) OCTA and vascular density map of choriocapillaris as heat map showing coarse pattern. 2b. Day 10 (Top) Fundus photos showing decreased areas of cotton wool spots.(Middle) Decreased sub-retinal fluid level but choroidal thickness increased. (lower and Bottom) Choriocapillaris signals and vascular density map returning to more fine signals but still with presence of flow voids. 2c. (Day 24)(Top) Normal fundus with sharp foveolar reflex. (Middle) Complete resolution of subretinal fluid with decreased macular thickness with normalization of choroidal thickness. (Lower and Bottom) OCTA and vascular density map of the choroid showing near normal choriocapillaris pattern.

Her visual acuities were 5/60 OD and 4/60 OS. The slit-lamp biomicroscopic evaluation of the anterior chamber was normal.Dilated fundus examination of RE showed clear vitreouswith hyperemic discwith blurred margin.Multiple whitish fluffy lesion extending over the superotemporal arcade and over macular area with formation of ‘macular star’ (Fig. 1a,Fig. 2a).Optical coherence tomography(OCT) and OCT-angiography (OCTA)were done usingTopcon Medical Systems‐Triton Deep Range Imaging(DRI) PLUS SS‐OCT which revealed thickened and detached neurosensory retina over the macular regionof both eyes.Additionally, OCTA of both eyesfor choroidal vasculature revealed abnormal “patchy” flow voids in the choriocapillaris-likely suggestive of a sluggish blood flow or ischemia. Deep range imaging (DRI) of the choroid revealed increased choroidal thickness and dilated choroidal vasculature, indicating a concurrent choroidal inflammation(Fig. 1a, b, Fig. 2a, b).

Baseline blood, biochemical, and serological investigations were done to rule out pathologies mimicking a similar clinical picture.Then, a working diagnosis of Post typhoid fever neuroretinitis with serous retinal detachment was made. The patient was started on oral prednisolone 1mg/kg/day for 10 days, oral ciprofloxacin 500mg twice daily for a week, topical flurbiprofen 0.03% four times a day, and topical tropicamide 1% twice a day.Adjunct antibioticswere added as the patient had defaulted the previously prescribed medication on day 7.She was advised to follow-up in 10 days.

At follow-up, the patient's visual acuity had improved to 6/24 in both eyes with no further improvement on refraction. The OCTA evaluationrevealedthat the central serous detachment had decreased with minimal residual subretinal fluid(Fig. 1, Fig. 2b). OCTA imaging of the retina was normal.Although the choroid's OCTA also revealed reduction in flow voids,DRI still revealed a persistent and increasedchoroidal thickness in both eyes(Fig. 1, Fig. 2b). Considering the possibility of an ongoing choroidal inflammation despite improvement in retinal imaging,the patient was advised to continue the steroid at the same dose for 10 more days while discontinuing the antibiotic. Topical medications were unchanged.

The next follow up was on day 24 after the initial presentation. The patient had vision of 6/12 and best corrected vision of 6/9 in BE. On OCT, the SRF had completely resolvedwith thinning of macular area – likely suggestive of atrophy.The choroidal thickness was normalizing with architecture of the choriocapillaris returning to near normal(Figs. 1c and 2c). Her vitals were stable. We tapered the steroid at the rate of 10mg/week while continuing the topical medication. She was advised to follow-up after 4 weeksfor visual field but was lost to follow up.

## 3 Discussions

In this case report, we described a patient with a recent history of typhoid fever who developed bilateral neuroretinitis with star-shaped maculopathy associated with bilateral serous detachment.

Prabhushanker et alreported a case of bilateral retinitis following typhoid fever.^4^ The fundus examination of the right eye showed white fluffy lesions and superficial hemorrhages around macula with macular star suggestive of retinitis. The OCT showed macular serous retinal detachment. The patient was treated with steroids and followed every 2 weeks for 3 months. The final OCT of the right eye revealed complete resolution of serous detachment. However,due to minimal disc edema, neuroretinitis was ruled out.

There are very few case reports on this rare condition. Retinal infiltration is the assumptive pathophysiology behind the condition.^5^, ^6^, ^7^ Neuroretinitis of other origins like cat scratch and Lyme's diseases has been reported more frequently.^8^^,^^9^ A pathology that incites immune-mediated response can disturb the blood-retinal barrier.^10^ It has been hypothesized that a micro emboli can obstruct microcirculation, eventually leading to sluggish blood flow and disc edema.^11^, ^12^, ^13^ A similar pathological cascade is thought to result in retinal vasculature leakage, causing exudative retinopathy.^14^

It has been also been postulated that immunological complexes are behind the pathogenesis of immune-mediated vasculitis secondary to typhoid infection. The complexes, in return, inflicts further damageby affecting self-antigens via homology or molecular mimicry.

Case reports by Laul R et al. and Relhan N et al. found that typhoid manifests as vasculitis and neuroretinitis with OCT revealing macular sensory detachment.Cases were successfully treatedwith oral steroids.^15^^,^^16^ However,the role of steroid has always been controversial in the treatment of infective neuroretinitis due to the lack of literature on this topic.^8^^,^^9^ Mild cases may show spontaneous resolution but severe cases require treatment.

In our case, oral ciprofloxacin and oral steroid werestarted on the day of presentation and baseline investigation was done to rule out other causes. On the fourth week of follow up, complete resolution of disc edema and SRF with faint impression of macular star and improved visual acuity were noticeable In addition to OCT, we used OCTA and choroidal imaging(DRI) to evaluate the status of both retina and choroid and to monitor the response to treatment. So far, many literatures havereportedonly about the insult to the optic disc and retina. Using DRI and OCTA of the choroid, we were able to report the involvement of the choroid.We also found that the inflammation of the choroid continued to persist even after the clinical signs suggested a resolution—such as decrease in the SRF and improvement of the visual acuity. OCTA which initially revealed a disrupted architecture of the choriocapillaris vasculature; gradually regained its much more “granular” pattern after initiating the treatment.This also further coincided with improvement in visual acuity.

Various reports have indicated that choroidal thickness and assessment of its vascularity can help in decision making and monitoring disease progression.^17^, ^18^, ^19^, ^20^ In our previous report of a sympathetic ophthalmia which had a similar clinical features in the posterior segment, we had expressed how choroidal thickness and its vasculature pattern/architecture analysis using OCTA could be helpful in monitoring the disease progression.^21^ The same imaging technique was found to be important in the management of this caseas well. This,we believe, further adds to the evidence that OCTA of the retina and the choroid and choroidal thickness have the potential to be used as vital indicators in monitoring the pathologies of the uvea and retina.

## 4 Conclusion

A rare case of noninfectious, immune-mediated, neuroretinitis and vasculitis with macular neurosensory detachment that occurred after the resolution of typhoid fever can be treated effectively with oral steroid. Noninvasive imaging modalities such as OCT, OCTA and DRI indicate choroidal involvement.These imaging techniques also play important roles in monitoring both the progression of the disease and the response to treatment.

## Funding/grant

No funding or grant support

## Conflict of interest

All of the authors do not have any conflicts of interest to declare.

## Ethical consideration/patient consent

Written consent to publish this case has not been obtained. This report does not contain any personal identifying information.

## Authorship

All authors attest that they meet the current ICMJE criteria for Authorship.

## References

[1] Pegeus D.A., Miller S.I., Fauci A.S., Braunwald E., Isselbacher K.J., Wilson J.D., Martin J.B., Kasper D.L. (2012). Salmonellosis. Harrison's Principles of Internal Medicine.

[2] Curtis T.H., Whealer D.T., Roy F.H., Fraunfelder F.W., Fraunfelder F.T. (2008). Infectious diseases. Current Ocular Therapy.

[3] Hughes E.H., Dick A.D. (2003). The pathology and pathogenesis of retinal vasculitis. Neuropathol Appl Neurobiol.

[4] Prabhushanker M., Tasmeen T., Topiwalla (2017). Bilateral retinitis following typhoid fever. Int J Retin Vitr.

[5] Thapar S. (2010). Characteristic OCT Patterns of Posterior Uveitis, Abstracts of All India Ophthalmology Conference (AIOC).

[6] Vishwanath S., Badami K., Sriprakash K.S., Sujatha B.L., Shashidhar S.D., Shilpa Y.D. (2013). Post-fever retinitis: a single center experience from south India. Int Ophthalmol.

[7] Fusco R., Magli A., Guacci P. (1986). Stellate maculopathy due to Salmonella typhi. Ophthalmologica.

[8] Brahm P.G., Sandeep K., Neha C. (2017 Mar). Neuroretinitis as presenting and the only presentation of Lyme disease: diagnosis and management. Indian J Ophthalmol.

[9] Ormerod L.D., Dailey J.P. (1999). Ocular manifestations of cat-scratch disease. Curr Opin Ophthalmol.

[10] Böke W.R.F., Manthey K.F., Nussenblatt R.B. (1992).

[11] Jaffe J S Strober, W Sneller M C Functional abnormalities of CD8+ T cells define a unique subset of patients with common variable immunodeficiency.Blood .82199319220. 8100719

[12] Chatzoulis D.M., Theodosiadis P.G., Apostolopoulos M.N., Drakoulis N., Markomichelakis N.N. (1997 May 1). Retinal perivasculitis in an immunocompetent patient with systemic herpes simplex infection. Am J Ophthalmol.

[13] Abu El-Asrar Ahmed M., Herbort Carl P., Tabbara Khalid F. (2005). Retinal vasculitis. Ocul Immunol Inflamm.

[14] Talat L., Lightman S., Tomkins-Netzer O., Ziehrut M. (Apr 15;2014). Ischemic retinal vasculitis and its management. J Ophthalmol. 2014.

[15] Laul R., Atif Ali M.I.R., Shafi S. (2015). Typhoid aftermath: presenting as vasculitis, neuroretinitis and macular neurosensory detachment. Int J Med Res Health Sci.

[16] Relhan N., Pathengay A., Albini T. (2014). A case of vasculitis, retinitis and macular neurosensory detachment presenting post typhoid fever. J Ophthalmic Inflamm Infect.

[17] Mahajan Sarakshi, Invernizzi Alessandro, Agrawal Rupesh, Biswas Jyotirmay, Rao Narsing A., Gupta Vishali (2017). Multimodal imaging in sympathetic ophthalmia. Ocul Immunol Inflamm.

[18] Agrawal Rupesh, Visva Gunasekeran Dinesh, Raje Dhananjay (2018). For the collaborative ocular tuberculosis study group; global variations and challenges with tubercular uveitis in the collaborative ocular tuberculosis study. Invest Ophthalmol Vis Sci.

[19] Mahajan S., Invernizi A., Agarwal R., Gupta V. (2016). Multimodal imaging in sympathetic ophthalmia. Ocul Immunol Inflamm.

[20] Agrawal R., Li L.K., Nakhate V., Khandelwal N., Mahendradas P. (2016). Choroidal vascularity index in vogt-koyanagi-harada disease: an EDI-OCT derived tool for monitoring disease progression. Transl Vis Sci Technol.

[21] Khatri A., Timalsena S., Khatri B.K. (2020). A rare entity: sympathetic ophthalmia presumably after blunt trauma to the phthisical eye and optical coherence tomography angiography metrics to monitor response to treatment. Clin Case Rep.

